# Anti-inflammatory and cytotoxic activities of the extracts, fractions, and chemical constituents isolated from *Luehea ochrophylla* Mart

**DOI:** 10.1186/s12906-019-2701-7

**Published:** 2019-10-28

**Authors:** Clináscia Rodrigues Rocha Araújo, Thiago de Melo Silva, Michaelle Geralda dos Santos, Marcelo Henrique Fernandes Ottoni, Elaine Maria de Souza Fagundes, Humberto de Sousa Fontoura, Gustavo Eustáquio Brito Alvim de Melo, Antônio Flávio de Carvalho Alcântara

**Affiliations:** 1 Departamento de Química, Instituto de Ciências Exatas, Universidade Federal de Minas Gerais, Belo Horizonte, Brazil; 2 Instituto Federal do Norte de Minas Gerais, Januária, Brazil; 3 Departamento de Farmácia, Universidade Federal dos Vales do Jequitinhonha e Mucuri, Diamantina, Brazil; 4 Departamento de Fisiologia e Biofísica, Instituto de Ciências Biológicas, Universidade Federal de Minas Gerais, Belo Horizonte, Brazil; 5 Departamento de Fisioterapia, Universidade Estadual de Goiás, Goiânia, Brazil

**Keywords:** *Luehea ochrophylla*, Triterpenes, Flavonoids, Cytotoxic activity, Anti-inflammatory activity

## Abstract

**Background:**

Stem bark of *Luehea ochrophylla* (*L. ochrophylla*) is used by the traditional Brazilian medicine for treatment of rheumatic diseases and tumors. This study aimed to investigate inhibition of acute and chronic inflammations and cytotoxic activity of extracts, fractions, and isolated compounds from *L. ochrophylla.*

**Methods:**

Hexane (HE) and ethanol (EE) extracts obtained from stem bark of *L*. *ochrophylla* were submitted to chromatographic fractionation. In order to test acute inflammation, experimental model of impact injury was used, followed by transdermal application of gels using phonophoresis. Histological analysis was based on scores assigned by the capacity of decreasing the lesion. To evaluate the effect EE and fractions on cell proliferation, human lymphocytes were stimulated with phytohemagglutinin and analyzed using flow cytometry. Proliferation was measured using VPD 450 staining and the calculated proliferative index (PI). The cytotoxic activity was evaluated using MTT colorimetric method against MDA-MB-231, MCF-7, HCT-116, and Vero cells. GraphPad Prism Version 5 was used for statistical analysis.

**Results:**

HE and EE provided friedelin, *β*-friedelinol, lupeol, mixture of lupeol and pseudotaraxasterol, *β*-sitosterol, betulinic acid, mixture of lupeol and taraxasterol, (−)-epicatechin, *β*-sitosterol-3-O-*β*-D-glucopyranoside, and (+)-epicatechin-(4*β*-8)-epicatechin. HE, ethyl acetate fraction (AF), betulinic acid, and *β*-sitosterol promoted regeneration of muscle fibers caused by muscle injury. AF significantly (*p* < 0.05) reduced the lymphocyte proliferation index (1.36 for cultures stimulated with PHA, 0.7 for untreated cultures and 0.12 for cultures stimulated with PHA and treated with AF 25 μg/mL and AF 50 μg/mL, respectively). *β*-Sitosterol-3-*O*-*β*-D-glucopyranoside exhibited high cytotoxic activity (IC_50_ = 1.279 μg/mL) against HCT-116 cell line.

**Conclusion:**

These results suggest that extracts, fractions, and chemical constituents from *L. ochrophylla* decreases inflammatory processes generated by muscle injury. The anti-inflammatory activity may be justified by high inhibition of T cell proliferation. These extracts, fractions, and chemical constituents from *L. ochrophylla* may be useful as a therapeutic agent against rheumatic diseases. Moreover, chemical constituents from *L. ochrophylla* show potent cytotoxic activity against colon and rectal carcinomas.

## Background

Inflammation is a response to tissue damage, which plays a role in the elimination of pathogens, neutralization of endotoxins and repair of injuries. The inflammatory response is characterized by increase in permeability of blood vessels, with migration of fluid, proteins, and leukocytes from circulation to the site of damage [1]. Eventually, inflammatory response can be exacerbated or weakly controlled, leading to extensive lesions of normal cells and tissue [2].

Innate immune system is the first line of defense of the organisms against pathogens and toxins. This system involves several strategies based on the recognition of molecular patterns deciphered by receptors that either induce or inhibit an immune response. Immune cells, such as neutrophils, dendritic cells, monocytes, and fully differentiated tissue-resident macrophages, are the primary cells involved in acute inflammation [3].

Lymphocytes are important cell populations involved in chronic inflammation. Those cells can regulate immune system, eliminate infected cells and orchestrate immune response by producing and secreting different kinds of cytokines. Cytokines, such as IL-2, TNF-α, and IFN-γ, are important in the progression of an inflammatory response aiming return to the homeostasis [4, 5].

Epidemiological studies show that inflammatory mediators and effector cells are important constituents of the local environment of tumors. Inflammation in the tumor microenvironment aids proliferation and survival of malignant cells, promotes angiogenesis and metastasis, subverts adaptive immune responses, and alters responses to hormones and chemotherapeutic agents [6].

*Luehea* (Malvaceae) is an American genus that is distributed across Mexico to Uruguay and Argentina [7]. Species of the genus *Luehea* are used in popular medicine to treat fungal infections [8], dysentery, leukorrhea, gangrenous wounds, and tumors [9].

In laboratory studies, an antiedematogenic effect was observed in the usage of aqueous extract from the leaves of *Luehea divaricata* in an animal model of carrageenan-induced edema [10]. Barks of *L. divaricata* presented antiedematogenic and analgesic effects in animals subjected to edema induced by carrageenan test, writhing, formalin and capsaicin tests [11]. Concerning anti-proliferative activity, the methanolic extract from the leaves of *L. candida* exhibited a strong inhibitory effect on the proliferation of human kidney cancer cells (line 786–0) [12]. The literature also describes antifungal action of the extract from *L. divaricata* bark on the growth inhibition of fungal dermatophytes such as *Microsporum canis*, *Trichophyton rubrum*, *Epidermophyton floccosum*, and *Microsporum gypseum*. The possible mode of action of those extracts against dermatophytes could be associated with the inhibition of polymers synthesis or cell wall assembly [13]. In addition, the methanol extract of *L. divaricata* showed antimicrobial activity against the *Micrococcus luteus* bacteria [14] and the ethanol extract of *L. paniculata* leaves showed inhibitory effect on *Staphylococcus aureus* growing [15].

*L. ochrophylla* is a tree popularly known in Brazil as “açoita-cavalo” and its barks are used to treat inflammations, rheumatism, arthritis, and tumors [16]. Literature does not describe a study confirming its popular use. Aiming to contribute to the understanding of possible biological properties of this species, the present work describes the effect of the plant extract on the proliferative response of human peripheral blood lymphocytes. The anti-inflammatory activity of the *L*. *ochrophylla* extracts, fractions and isolated compounds was investigated by transdermal application in rats after induction of muscle injury. The cytotoxic activity was also assessed against breast carcinoma lines (MDA-MB-231 and MCF-7) and carcinoma of colon and rectum (HCT-116).

## Methods

### General procedures

Phosphate buffered saline (PBS; pH 7.2), Ficoll-Histopaque, phytohemagglutinin (PHA), RPMI-1640 medium, L-glutamine, antibiotic/antimycotic cocktail, trypan blue, ketamine, xylazine, 3-bromide (4,5-dimethyltiazol-2-yl)-2,5-diphenyltetrazolium (MTT), and dimethyl sulfoxide (DMSO) were purchased from Sigma-Aldrich (St. Louis, MO, EUA). Heat-inactivated fetal calf serum (FCS) was purchased from Gibco (Invitrogen Corporation, Grand Island, NY, EUA). BD Horizon™ Violet Proliferation Dye 450 (VPD450) was purchased from BD Biosciences (San Jose, CA, EUA). Dexamethasone at 8 μg/mL and dexamethasone cream 0.1% were purchased from Aché (Guarulhos, São Paulo, Brazil) and Teuto (Anápolis, Goiás, Brazil), respectively.

Uncorrected melting points were determined using METTLER equipment, model FP80 SNR H22439. IR spectra were taken on Perkin Elmer Spectrum One (ATR) and Shimadzu FTIR-8400 (KBr pellets) spectrometers. ^1^H and ^13^C NMR spectra, including the ^1^H-^1^H COSY, ^1^H-^1^H NOESY, ^1^H-^13^C HMBC, and ^1^H-^13^C HSQC experiments, were performed on Bruker DRX 400 and Bruker DPX 200 AVANCE spectrometers, using CDCl_3_, C_5_D_5_N, CD_3_OD, or DMSO-*d*_6_ as solvent. The chemical shifts were measured in parts per million (*δ*) relative to TMS which was used as internal standard. The coupling constants (*J*) were recorded in Hertz. Silica gel 60 (F254) was used for analytical thin layer chromatography (TLC) and silica gel 60 (70–230 mesh) was used for column chromatography (CC). Spots on chromatograms were detected under exposure to UV light (254 and 365 nm) and by spraying with vanillin/H_2_SO_4_ solution followed by warming. Optical rotation values [α]_D_^20^ were measured on a Bellingham & Stanley ADP-220 polarimeter.

### Phytochemical methodology

#### Plant material

Stem barks of *L. ochrophylla* were collected in August 2012 in the City of Esmeraldas, State of Minas Gerais (Brazil). A voucher specimen of *L. ochrophylla* has been deposited at the Dendrological Herbarium Jeanine Felfili on the Universidade Federal dos Vales do Jequitinhonha e Mucuri (UFVJM), registered as HDJF2043. The plant was identified by A. Riguetti Corrêa (The National School of Tropical – Botanical Garden Research Institute - RJ). The stem barks of the plant were dried at room temperature until constant weight was achieved (about 1 week) and it was finally powdered.

#### Extraction and isolation

The powdered plant material (4400.00 g) was successively subjected to maceration with hexane and ethanol at room temperature, providing the crude hexane (HE; 8.30 g) and ethanol (EE; 153.46 g) extracts, after concentration under reduced pressure. An aliquot of HE (7.0 g) was submitted to column chromatography using hexane, chloroform, and ethanol, in the order of increasing polarity. An aliquot of EE (140.2 g) was submitted to column chromatography using dichloromethane, ethyl acetate, and ethanol, providing fractions DF, AF, and EF, respectively. DF (3.2 g) and AF fractions were rechromatographed [17].

### Anti-inflammatory tests

#### Animals

Experiments were performed on male rats (200.0 to 300.0 g) purchased from Bioagri Laboratories LTDA (Planaltina, Distrito Federal, Brazil). Animals were kept in plastic cages at (22 ± 2) °C on a 12 h light/dark cycle with free access to pellet food and water, according to International Guiding Principles for Biomedical Research Involving Animals. The animals were acclimatized for 4 days before beginning the experiments. The entire experiment was approved by the Ethics Committee on Animal Use of the Universidade Federal de Minas Gerais, under protocol number 221/11. All sections of this study were adhered to the ARRIVE Guidelines for reporting animal research.

#### Muscle injury and treatment

Initially, ketamine and xylazine (80.0 and 10.0 mg/kg body weight, respectively) were diluted in 1.00 mL of saline solution and employed to intraperitoneal anesthesia. Animals were anesthetized and suffered muscle injury by the impact of a loose weight of 300.0 g at 30.0 cm high in both paws (right and left). Animals were separated into groups (*n* = 3 for each group): a negative control group (without treatment), a positive control group (animals treated with dexamethasone 0.1%), and six groups with treatment (animals treated with samples of HE, AF, betulinic acid, *β*-sitosterol, (−)-epicatechin or *β*-sitosterol-3-*O*-*β*-D-glucopyranoside). HE (6.0% m/m), AF (3.0% m/m), isolated compounds (0.5% m/m), and dexamethasone (DEXA) were incorporated in the form of carbopol gel [18].

After muscle injury, trichotomy was performed on bilateral gluteal region of both injured paws. The right paw of each animal was treated by application of gels previously prepared and using therapeutic ultrasound in pulsed mode at a frequency of 1 MHz, with intensity 0.5 W/cm^2^ for 9 min [19]. The left paw of each animal was used as negative control. After 24 h on treatment, animals were anesthetized and sacrificed by cervical spine dislocation. Then the isquiotibial muscle was collected. Other similar sacrifices were repeated after 48 and 72 h. The paw tissues were removed, fixed in 10% formalin phosphate buffered saline (PBS) embedded in paraffin, and cut into 4 μm thickness sections. The sections were stained by hematoxylin-eosin for histological analysis. A representative area was selected for qualitative light microscopic analysis of the inflammatory cellular response with a 10X objective [20]. The results were expressed by the ability to decrease the inflammatory infiltrate displayed in the images. The decrease of the interstitial space (the blank space in the images) coincided with the reduction of the inflammatory infiltrate.

### Antiproliferative activity of lymphocytes

#### Peripheral blood mononuclear cells (PBMC) obtention

Peripheral blood was obtained from five healthy adult volunteers, which did not use any medicine in the week before. Heparinized human peripheral blood was diluted with PBS and centrifuged in Ficoll-Histopaque (400×g at room temperature for 30 min) for obtaining the characteristic layer containing mononuclear cells [21]. PBMC were collected, washed with PBS, and centrifuged (240×g at room temperature for 7 min). Cells were suspended at 1 × 10^7^ cell/mL in PBS or RPMI-1640 medium supplemented with 10% FCS, 2 mM L-glutamine, 100 UI/mL penicillin G, 100 μg/mL streptomycin and 250 ng/mL amphotericin B. PBMC from each research subject were separately obtained and individually used for cell culture analysis. The study was approved by the Ethical Committee at the UFVJM (register code 569.313/2014) and a written consent was informed by each volunteer.

#### Cell viability analysis

PBMC (5 × 10^5^ cells/ml) were cultured with 0.5% DMSO (solvent control), EE (100, 50, or 25 μg/mL), AF (200, 100, or 50 μg/mL), or EF (100, 50, or 25 μg/mL) at 37 °C in a humidified incubator with a 5% CO_2_ atmosphere for 24 h or 5 days. As HE extract and DF fraction were not soluble in DMSO, they were not tested. Untreated PBMC was used as the unstimulated cell culture control (Ctrl). After incubation period, cells were washed with PBS and stained with 0.4% trypan blue. Total, viable, and non-viable cells were counted in an optical microscope by using a Neubauer chamber.

#### Lymphocyte proliferative response

PBMC (1 × 10^7^ cells) was suspended in PBS and labeled with 1 μM of VPD450 for 15 min at 37 °C [22]. VPD 450-stained PBMC (5 × 10^5^ cells/well in 24-well plate) were cultured in RPMI-1640 supplemented with 10% FCS, 2 mM L-glutamine, 100 UI/mL penicillin G, 100 μg/mL streptomycin and 250 ng/mL amphotericin B, with or without PHA (5 μg/mL). Cells were also stimulated with PHA in combination with 0.5% DMSO (solvent control) or concentrations of samples of EE, AF, and EF, among the testing concentrations in the cell viability analysis (EE 25, 15, or 7.5 μg/mL, AF 100, 50, or 25 μg/mL, and EF 50, 25, or 15 μg/mL) which do not promote death of human PBMC. Cells stimulated with PHA in combination with DEXA at 8 μg/mL were used as the inhibition control. The plate was kept in a humidified incubator with a 5% CO_2_ air atmosphere for 5 days at 37 °C. Samples were analyzed on a BD FACSCanto™ II with BD FACSDiva™ software, and 50,000 events were acquired for each tube. Proliferation was measured based on the reduction of VPD 450 fluorescence intensity resulted of cell division. The proliferative index (PI) was then calculated based on the violet fluorescence histograms by using the formula: PI = (100 − Y)/Y, where Y (%) = X_0_ + X_1/2_ + X_2/4_ + X_3/8_ + X_4/16_ + X_5/32_ + X_6/64_. X_0_ corresponds to percentage of T-cells that did not divide (located in M1), and X_1_-X_6_ represents the peaks of gradual division (located in M2-M7) [23].

### Cytotoxicity assay

Stock solutions (20 mg/mL in DMSO) were prepared with extracts, fractions or isolated compounds for cytotoxicity assays. The samples were dissolved just before the experiments, to minimize the variability among the tests and/or instability of the samples. The final concentration of extracts, fractions, and compounds in screening was 30 μg/mL, with a maximum concentration of DMSO of 0.5%, avoiding solvent interference on cell viability. Human breast cancer cell lines (MCF-7 and MDA-MB-231), human colon and rectal cancer cell line (HCT-116), and non-tumorigenic cell line (Vero) were used. All cell lines were maintained in logarithmic phase growth in RPMI-1640 supplemented with 100 U/mL of penicillin, 100 μg/mL of streptomycin, 2 μmol/L of L-glutamine, and 10% fetal calf serum. Cells were added to the wells of a microplate at a density of 10,000 cells and incubated for 24 h at 37 °C and 5% CO_2_. After stabilization, cells were incubated with samples (30 μg/mL) for 48 h at 37 °C and 5% CO_2_. A negative control containing the solvent (DMSO) and positive control (cisplatin) were run in the same concentration of the samples. Two independent experiments were carried out in triplicate. Samples that inhibited more than 50% cell viability were subjected to determination of the half maximal inhibitory concentration (IC_50_) for serial dilution ranging from 100.0 to 1.5 μg/mL. Cell proliferation was measured based on metabolic reduction of MTT to formazan [24]. Briefly, 20 μL of MTT solution (2 mg/mL) were added to each well 4 h before the end of incubation period. After 4 h, formazan crystals were observed and supernatant was carefully removed. To each well, 200 μL of HCl solution (0.04 M) in isopropanol were added. After solubilization of the formazan crystals, absorbance of the solution was read in ELISA reader at a wavelength of 595 nm. For calculation of the percentage of cell viability inhibition, it was used the formula: % inhibition = 1 - (Absorbance of the sample × 100) / Absorbance of the control (DMSO). Data were expressed as IC_50_ to extracts, fractions, and isolated compounds that inhibited more than 50% of the cell viability.

### Data analysis

GraphPad Prism, version 5.0 for Windows (GraphPad Software, USA), was used for statistical analysis, and *p*-values < 0.05 were considered to be statistically significant. Data are reported as mean ± standard deviation (SD). Analysis of variance (one-way ANOVA) was employed, followed by Tukey’s post hoc pairwise comparisons.

## Results

### Phytochemical study

**Fig. 1:**
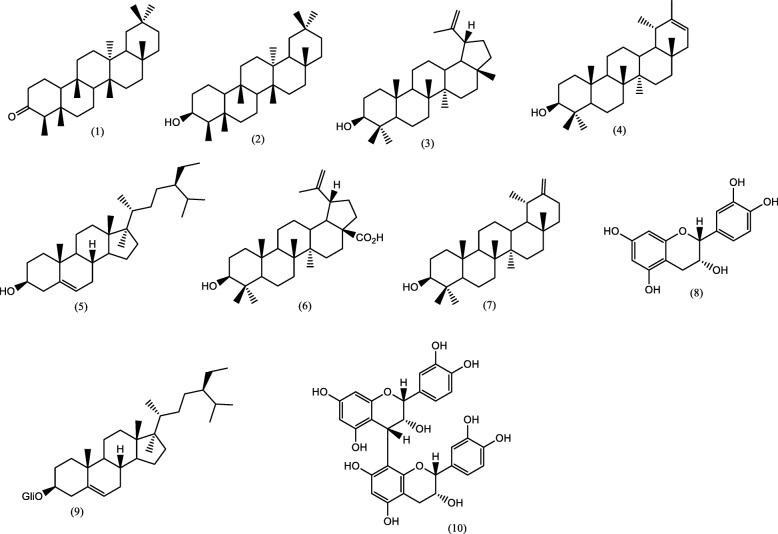
Chemical structure of the compounds isolated from stem bark of *L*. *ochrophylla* Mart: Friedelin (**1**), *β-*friedelinol (**2**), lupeol (**3**), pseudotaraxasterol (**4**), *β*-sitosterol (**5**), betulinic acid (**6**), taraxasterol (**7**), (−)-epicatechin (**8**), *β*-sitosterol-3-*O*-*β*-D-glucopyranoside (**9**), and (+)-epicatechin-(4*β*-8)-epicatechin (procyanidin B2) (**10**)

Phytochemical study of HE provided friedelin, *β*-friedelinol, lupeol, mixture of lupeol and pseudotaraxasterol, *β*-sitosterol, and betulinic acid. DF fraction provided a mixture of lupeol and taraxasterol. AF fraction provided (−)-epicatechin, *β*-sitosterol-3-*O*-*β*-D-glucopyranoside, and (+)-epicatechin-(4*β*-8)-epicatechin (procyanidin B2) as shown in Fig. 1.

### Anti-inflammatory tests

#### Transdermal drug application

**Fig. 2:**
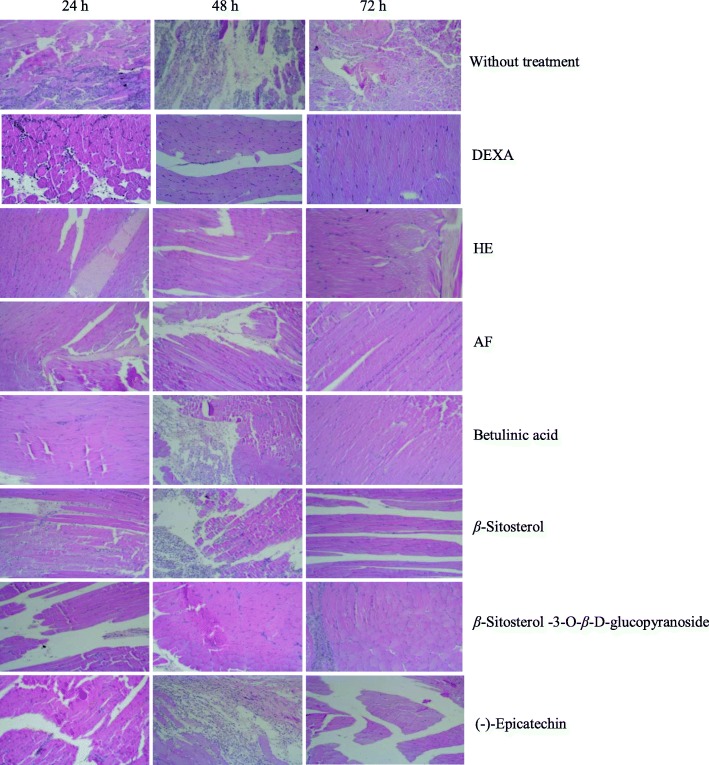
Histological aspect of the injured muscles of rat paws without treatment and treated for 24, 48, and 72 h with the application of gels with dexamethasone, hexane extract (HE), ethyl acetate fraction (AF), betulinic acid, *β*-sitosterol, *β*-sitosterol-3-O-*β*-D-glucopyranoside, and (−)-epicatechin. 10x magnification

Evaluating the activity of HE, AF and compounds isolated from stem bark of *L*. *ochrophylla* on an acute inflammation model, it was performed histological analysis for the characterization of lymphocytic inflammatory infiltrates, edema, and degeneration of muscle fibers. Figure 2 shows histological aspects of the injured muscle of rat paw without treatment (negative control group) after 24 h of the muscle injury. The section exhibits edemas (white regions), inflammatory infiltrates (purple dots spread on the section), and fragmentations of muscle tissue (discontinuous pink spots). Figure 2 also shows histological aspects of the injured muscles of the rat paws treated with DEXA (positive control group), HE, AF, and compounds *β*-sitosterol, betulinic acid, (−)-epicatechin, and *β*-sitosterol-3-*O*-*β*-D-glucopyranoside.

After 48 and 72 h, negative control group presented a considerable increase in the inflammatory infiltrate and edema. Positive control showed a mild inflammatory infiltrate after 24 and 48 h of muscle injury, although it is possible to verify regions of edema. After 72 h, the histological section shows an area of significant regeneration of muscle fibers and few regions of edema and inflammatory infiltrate. HE and AF showed considerable anti-inflammatory activity after 24, 48, and 72 h of treatment. After 72 h of treatment there was almost a complete regeneration of muscle fibers, absence of inflammatory infiltrate and edema. Such data can be similar to the data of the positive control.

*β*-Sitosterol and betulinic acid, isolated from HE, have anti-inflammatory activity observed by the regeneration of muscle fibers and reduction of inflammatory infiltrate after 72 h of treatment. *β*-Sitosterol-3-*O*-*β*-D-glucopyranoside exhibits anti-inflammatory activity, promoting a reorganization of muscle fibers and decreasing edema after 72 h of treatment. After this time, it is still possible to verify inflammatory infiltrates. (−)-Epicatechin showed little anti-inflammatory activity. After 48 h of treatment it was observed intense inflammatory infiltrate and after 72 h of treatment it was still observed a significant edema region.

### Antiproliferative activity of lymphocytes

#### Cell viability analysis

For in vitro assays, cell viability assays were first performed to determine the non-cytotoxic concentrations. After 24 h of culture, it was verified that concentrations ≤50.0 μg/mL of EE and EF and concentrations ≤100.0 μg/mL of AF did not reduce PBMC viability when compared to control and solvent cultures (Table 1). After 5 days of culture, it was verified that concentrations ≤15.0 μg/mL of EE, concentrations ≤25.0 μg/mL of EF, and concentrations ≤50.0 μg/mL of AF had do not decreased PBMC viability, when compared to control and solvent cultures (Table 2).

**Table 1:** Effect of EE extract and AF and EF fractions from stem bark of *L*. *ochrophylla* on cell viability after 24 h of culture

	Cell viability after 24 h		
	μg/mL	Mean	SD
EE	25.0	97.17^a^	1.33
EE	50.0	89.17^a^	6.59
EE	100.0	73.00^b^	7.24
AF	50.0	96.33^a^	1.63
AF	100.0	95.00^a^	1.09
AF	200.0	77.83^b^	7.55
EF	25.0	98.50^a^	0.58
EF	50.0	93.33^a^	3.14
EF	100.0	81.00^b^	6.75
CTRL		98.00^a^	1.09
DMSO		97.67^a^	0.82

Values are expressed as mean ± standard deviation. Percentages of viable PBMC were assessed by using the trypan blue exclusion test (*n* = 8). Different letters in the same column indicate significant difference when (*P* < 0.05) after one-way ANOVA test with Tukey’s post hoc

**Table 2:** Effect of EE extract and AF and EF fractions from stem bark of *L*. *ochrophylla* on cell viability after 5 days of culture

	Viability after 5 days		
	μg/mL	Mean	DP
EE	7.5	91.17^a^	3.25
EE	15.0	92.67^a^	2.58
EE	25.0	72.33^b^	10.89
AF	25.0	87.17^a^	5.27
AF	50.0	82.17^a^	5.71
AF	100.0	54.17^b^	13.89
EF	15.0	90.17^a^	3.87
EF	25.0	81.33^a^	12.53
EF	50.0	60.17^b^	16.64
CTRL		92.17^a^	3.19
DMSO		92.67^a^	2.58

Values are expressed as mean ± standard deviation. Percentages of viable PBMC were assessed by using the trypan blue exclusion test (*n* = 8). Different letters in the same column indicate significant difference when (*P* < 0.05) after one-way ANOVA test with Tukey’s post hoc

Therefore, the concentrations 15.0 and 7.5 μg/mL of EE, 50.0 and 25.0 μg/mL of AF, and 25.0 and 15.0 μg/mL of EF were chosen for PBMC in vitro experiments.

#### Lymphocyte proliferative response

**Fig. 3:**
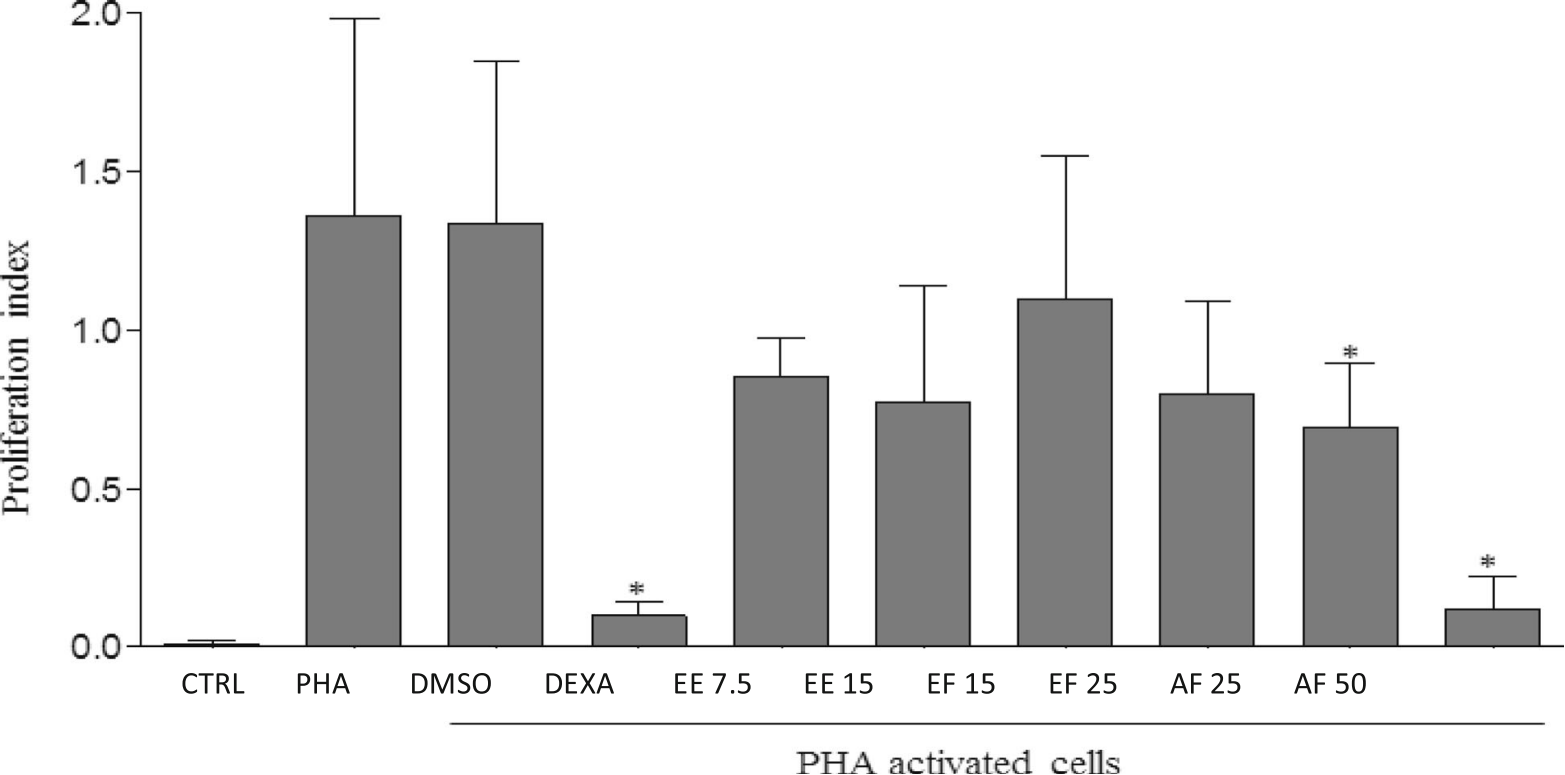
Influence of EE extract and EF and AF fractions on the proliferation of activated lymphocytes. VPD 450-labeled human lymphocytes (5 × 10^5^ cells) were cultured in the presence of medium, dexamethasone (DEXA, 8 μg/mL) or different concentrations of EE (7.5 and 15 μg/mL), EF (15.0 and 25 μg/mL), AF (25.0 and 50.0 μg/mL), and activated with PHA (5 μg/mL) for 5 days. The lymphocyte proliferation was analyzed using flow cytometry. The results are expressed as the mean ± SD of the proliferation index of human lymphocytes (*n* = 6). The ANOVA statistical method was used, followed by Tukey’s post hoc. * means significant difference (*p* < 0.05)

Effects of EE, AF, and EF of stem bark of *L*. *ochrophylla* on the proliferative response of lymphocytes are shown in Fig. 3 and Table 3. Different concentrations of EE (7.5 and 15.0 μg/mL) and EF (15.0 and 25.0 μg/mL) did not reduce lymphocytes proliferation index compared to cultures stimulated with PHA. However, AF fraction at concentrations of 25.0 and 50.0 μg/mL significantly reduced lymphocyte proliferation index, as well as dexamethasone (inhibition control). There was no change in cell proliferation when they were treated with DMSO, suggesting that the observed reduction of proliferation was exclusively due to the presence of AF in the cultures.

**Table 3:** Effect of EE extract and AF and EF fractions from stem bark of *L*. *ochrophylla* on the proliferation of activated lymphocytes for 5 days

	Proliferation of lymphocytes index (PI)		
	μg/mL	Mean	DP
EE	7.5	0.85^a^	0.12
EE	15.0	0.77^a^	0.37
AF	25.0	0.70^b^	0.20
AF	50.0	0.12^b^	0.10
EF	15.0	1.10^a^	0.45
EF	25.0	0.80^a^	0.30
Dexamethasone	8.0	0.10^b^	0.04
PHA	5.0	1.36^a^	0.62
Ctrl		0.01^b^	0.01
DMSO		1.34^a^	0.51

Values are expressed as mean ± standard deviation. Different letters in the same column indicate significant difference when (*P* < 0.05) after one-way ANOVA test with Tukey’s post hoc

### Cytotoxicity assay

Among the extracts (HE and EE), fractions (DF, AF, and EF) and tested compounds (friedelin, lupeol, *β*-sitosterol, *β*-sitosterol-3-*O*-*β*-D-glucopyranoside, and (−)-epicatechin) only lupeol and *β*-sitosterol-3-*O*-*β*-D-glucopyranoside inhibited more than 50% of the cancer cells lines growth used in the screening. Both compounds had IC_50_ determined. The results for lupeol and *β*-sitosterol-3-*O*-*β*-D-glucopyranoside cytotoxicity assay on MDA-MB-231, MCF-7, HCT-116 and Vero cells are presented in Table 4.

**Table 4:** In vitro effect of lupeol and *β*-sitosterol-3-O-*β*-D-glucopyranoside on the proliferation of human cancer cell lines and non-tumorigenic cell line (Vero cells)

Compounds	MDA-MB-231		MCF-7		HCT-116		Vero
	IC_50_ ± SD	SI	IC_50_ ± SD	SI	IC_50_ ± SD	SI	IC_50_ ± SD
Lupeol	60 ± 17.2	0.8	67 ± 22.4	0.8	35.2 ± 13.2	1.5	51 ± 18.2
*β*-Sitosterol-3-O-*β*-D-glucopyranoside	33 ± 10.1	0.6	11 ± 5.2	1.9	1.279 ± 0.7	16.4	21 ± 9.9
Cisplatin	> 100		> 100		> 100		13.05 ± 0.6

Data are presented as mean ± SD. *IC50* 50% inhibition of cell growth. *SI* Selectivity index calculated as follows IC50 (μg/mL) for Vero / IC50 (μg/mL) for tumor cells

*β*-Sitosterol-3-*O*-*β*-D-glucopyranoside and lupeol exhibited greater cytotoxicity to the tested tumor lines when compared to cisplatin (positive control). However, only *β*-sitosterol-3-*O*-*β*-D-glucopyranoside showed a high selectivity index (>2) to HCT-116 cells. Low selectivity index values (SI < 2) are indicative of toxicity [25].

## Discussion

### Transdermal drug application

The result of the transdermal application of *β*-Sitosterol and betulinic acid, isolated from HE, suggest that the anti-inflammatory activity of HE can be a result from synergistic effects among these compounds and other constituents of the extract.

The literature reports anti-inflammatory activity of betulinic acid by suppression of leukocytes and decreasing of levels of exudates at the site of inflammation in model of induced pleurisy with carrageenan [26]. Such anti-inflammatory effects presented by betulinic acid are justified by inhibiting the release of pro-inflammatory mediators, mainly NO, IL-1β, TNF-α, and reduction of COX-2 levels [26, 27]. *β*-Sitosterol exhibits anti-inflamatory activity by inhibiting ear edema induced by xylene [28], besides to reduce migration of leukocyte, neutrophils and the activity of myeloperoxidase and adenosine deaminase enzymes [29]. The literature reports also anti-inflammatory activity in vivo for other HE constituents, lupeol and taraxasterol. Lupeol reduces edema, degeneration of muscle fibers and the inflammatory infiltrate within 72 h of treatment [18]. Taraxasterol had efficacy comparable to prednisolone (a widely known anti-inflammatory) in a paw edema model induced by formalin [30].

*β*-Sitosterol-3-*O*-*β*-D-glucopyranoside showed inhibitory effect for the release of NO [31].

The anti-inflammatory activity of AF can also be justified by the presence of procyanidin B2, dimer of (−)-epicatechin. Although not individually tested in our experiment, literature reports that this compound shows high anti-inflammatory activity. Its activity in mouse ear model was higher than that presented by indomethacin and glycyrrhetinic acid [32]. Those data suggest high anti-inflammatory activity observed for AF in vivo can be considered as a synergistic effect of all of its constituents.

### Lymphocyte proliferative response

AF fraction at concentrations of 25.0 and 50.0 μg/mL significantly reduced lymphocyte proliferation index just like the reduction verified for the pharmacological dose of DEXA, used as an inhibition control. AF is constituted mainly by the flavonoid (−)-epicatechin and procyanidin B2, besides the steroid *β*-sitosterol-3-*O*-*β*-D-glucopyranoside. The significant inhibition of lymphocyte proliferation by AF may be related to flavonoids that inhibit the proliferation by decreasing IL-2 levels in cell culture [33]. Epicatechin modulates the specific immune response by inhibiting the activation of T lymphocytes and significantly reduce the IL-2 secretion and IL-2Rα expression (CD25), which is a subunit of its receptor [34]. The anti-inflammatory activity of procyanidin B2, also present in AF, has been thoroughly investigated. This dimer has a high anti-inflammatory activity by inhibiting, after lipopolysaccharide challenge, TNF-α and IL-6, besides increase IL-1 receptor expression associated with protein kinase (IRAK-M) and thus, it reduces inflammatory cytokines production. Procyanidin B2-treated macrophages inactivated naïve T cells through IRAK-M [35]. Another study reports that procyanidin B2 inhibits COX-2 expression with mechanism mediated by inhibition of MAPK protein phosphorylation and prevention of binding to DNA of NF-ĸB through stabilization of IĸB proteins [36]. Procyanidin B2 also inhibits the activation of the intracellular signaling of NLRP3 via suppression of AP-1 complex in endothelial cells. NLRP3 complex catalyzes cleavage of interleukin-1β precursor (IL-1b) and IL-18, pro-inflammatory cytokines involved in the host response to infection and tissue injury, being the unregulated activation NLRP3 involved in the pathogenesis of many diseases including atherosclerosis, heart disease and diabetes [37]. *β*-Sitosterol-3-*O*-*β*-D-glucopyranoside shows moderate inhibition of NO production [31].

### Cytotoxicity assay

*β*-Sitosterol-3-*O*-*β*-D-glucopyranoside can be considered as a strong cytotoxic agent against colon and rectal carcinomas, exhibiting low toxicity to normal cells. Cytotoxic activity of steroidal glycosides has been ascribed to the capacity of aglycone portion to form a system with cholesterol in biomembranes, while the sugar portion (hydrophilic) remains outside limiting cell where it interacts with glycolipids and glycoproteins. As a consequence, pores are formed in the biomembrane, which makes it permeable and susceptible to apoptosis [38].

Stem bark of *L*. *ochrophylla* presents cytotoxic activity against colon and rectum cancer cells HCT-116. Cytotoxic activity against the HCT-116 cell line observed for *β*-sitosterol-3-*O*-*β*-D-glucopyranoside is important because solid tumors are extremely resistant to chemotherapeutic agents currently available. Also, combinations of conventional chemotherapies have limited efficacy and are associated with significant toxicity [39].

Although other studies are required to confirm the action of *β*-sitosterol-3-*O*-*β*-D-glucopyranoside and its mechanism of action, the results presented here are biologically important. Colon and rectum cancer are frequent in malignancies neoplasias in the whole world. In Brazil, this cancer is the fourth leading cause of deaths, and about half of patients die within 5 years after the start of treatment [40].

## Conclusions

The results in the present study reveal the presence of compounds of different chemical classes, mainly steroids, triterpenes, and flavonoids. Topical application of extracts e compounds extracted from the stem bark of *L. ochrophylla* decreases inflammatory processes generated by muscle injury. *L. ochrophylla* significantly reduced lymphocyte proliferation index *p* < 0.05 and showed high cytotoxic activity against colon and rectal carcinoma cells (HCT-116 cell line). *L. ochrophylla* is a promising source of new effective phytomedicines, which, in the future, may be used for the treatment of injuries and different inflammatory disorders, so as to support in the elimination of colon and rectum carcinoma cells.

## Data Availability

The data was included in the figures of the manuscript. The supporting materials can be obtained upon request via email to the corresponding author.

## Abbreviations

AF: Acetate fraction
CC: Column chromatography
DEXA: Dexamethasone
DF: Dichloromethane fraction
EE: Ethanol extract
EF: Ethanol fraction
FCS: Inactivated fetal calf serum
HCT-116: Human colon and rectal cancer cell line
HE: Hexane extract
IC_50_: Half maximal inhibitory concentration
IR (ATR): Attenuated total reflection infrared spectroscopy
MCF-7: Human breast cancer cell line
MDA-MB-231: Human breast cancer cell line
MTT: 3-bromide (4,5-dimethyltiazol-2-yl)-2,5-diphenyltetrazolium
NMR: Nuclear magnetic resonance
PBMC: Peripheral blood mononuclear cells
PBS: Phosphate buffered saline
PHA: Phytohemagglutinin
PI: Proliferative index
RPMI-1640: Roswell Park Memorial Institute (cell culture medium)
TLC: Layer chromatography
Vero cells: African green monkey kidney non-tumorigenic cells
VPD450: Violet Proliferation Dye 450
